# Supplementary material for: Safety Concerns Related to the Simultaneous Use of Prescription or Over-the-Counter Medications and Herbal Medicinal Products: Survey Results among Latvian Citizens
Source: Int J Environ Res Public Health. 2023 Aug 9;20(16):6551. doi: 10.3390/ijerph20166551 (PMC10454617; doi:10.3390/ijerph20166551)
Supplement: Supplementary file 1 [file ijerph-20-06551-s001.zip › S1. Questionnaire herbal medicine.pdf]

Questionnaire

Dear participant, this questionnaire is a part of research carried out in Red Cross Medical College of Rīga Stradiņš University about the use of drugs and herbal medications. Your participation is confidential. All the data will be published only as a summary of responses.

This questionnaire is aimed for people who purchase their own medicine and have been using it for the past year. By filling in this questionnaire you confirm your participation in the research. Participation is voluntary. The questionnaire will take around 15 mins. You can receive clarification from the specialist who provided you with this questionnaire.

Thank you for your time!

General questions

1. **Sex**
   ☐ Female ☐ Male
2. **Age**
   ______
3. **Address**
   ☐ City (republic city: Rīga, Rēzekne, Daugavpils, Jēkabpils, Jelgava, Jūrmala, Liepāja, Valmiera, Ventspils)
   ☐ City (regional city - name)
   ☐ Country (regional parish - name)
4. **Cultural and historical region of Latvia**
   ☐ Vidzeme ☐ Latgale
   ☐ Zemgale ☐ Kurzeme
5. **Education**
   ☐ Primary education (general - grades 1 to 9, and professional)
   ☐ Secondary education (general, vocational, professional secondary, professional continuing education)
   ☐ Higher education (college, professional and academic Bachelor's degree, Master's degree)
   ☐ PhD

Factor 1 Chronic illness

6. **Do you have any chronic illness (long-term or recurring)**
   ☐ Yes
   ☐ No

7. **What illness or health problems have you had in the past year?** ***Multiple answers possible***

1.	Heart conditions	☐ Yes	☐ No
2.	Hypertension	☐ Yes	☐ No
3.	Lung disease	☐ Yes	☐ No
4.	Diabetes	☐ Yes	☐ No
5.	Stomach ulcer or any other disease of the gastrointestinal tract	☐ Yes	☐ No
6.	Kidney and urinary tract disease	☐ Yes	☐ No
7.	Liver disease	☐ Yes	☐ No
8.	Anaemia or other blood disease	☐ Yes	☐ No
9.	Cancer	☐ Yes	☐ No
10.	Depression, anxiety	☐ Yes	☐ No
11.	Arthritis of any kind	☐ Yes	☐ No
12.	Back pain	☐ Yes	☐ No
13.	Osteoporosis (bone atrophy)	☐ Yes	☐ No
14.	Allergies	☐ Yes	☐ No
15.	High cholesterol, hyperlipidemia	☐ Yes	☐ No
16.	Other medical condition	☐ Yes	☐ No

If yes (16), please specify ___________________

17. No health conditions □

8. Please indicate how many acute (sudden, severe, rapidly developing) health conditions you have had in the past year?

- ☐ None
- ☐ 1
- ☐ 2
- ☐ 3
- ☐ 4
- ☐ 5 and more
- ☐ Cannot remember

9. Have you experienced any medication side effects?

- ☐ Yes
- ☐ No

<u>Factor 2 Use of medication</u>

10. Have you used prescription medications?

- ☐ No
- ☐ Yes, during acute health conditions (sudden, severe, rapidly developing)
- ☐ During chronic illness
  - ➢ daily,
  - ➢ In the past month (30 days)
  - ➢ In the past three months (90 days)
  - ➢ In the past half a year (6 months)
  - ➢ In the past year (12 months)
  - ➢ As a course of medication

  (Multiple answers possible in case of the use of several medications)

10 a. Please indicate the most used prescription medication that has been used for both acute and chronic illness

- ☐ Please write the name of the medication____
  ____
  ____
  ____
  ____
  ____
- ☐ Do not remember the name

10 b. How many active ingredients of prescription medication are used at the same time? (Team of researchers to count separately for each questionnaire based on the name of the medication provided and their active ingredients)

1 2 3 4 5 6 7 8 9 10 11 12 13 14 15<

11. Have you used non-prescription medication?

- ☐ No
- ☐ Yes, during acute health conditions (sudden, severe, rapidly developing) or
- ☐ As a course of medication
- ☐ Use regularly
  - ➢ daily,
  - ➢ In the past month (30 days)
  - ➢ In the past three months (90 days)
  - ➢ In the past half a year (6 months)
  - ➢ In the past year (12 months)
  - ➢ Over a year

  (Multiple answers possible in case of the use of several medications)

11 a Please name the most used non-prescription medications

- ☐ Please write the name of the medication____
  ____
  ____

☐ Do not remember the name

11 b How many active ingredients of non-prescription medication are used at the same time? (Team of researchers to count separately for each questionnaire based on the name of the medication provided and their active ingredients)

1 2 3 4 5 6 7 8 9 10 11 12 13 14 15<

Factor 3 Use of herbal medication

12. Do you use herbal medication in your daily life? (in the form of teas, oils, tinctures, extracts, tablets, capsules or syrups)

☐ No, have never used
☐ No, have used but decided to discontinue
☐ No, have used but stopped based on doctor's recommendations
☐ No, have used but stopped due to other reasons
☐ Use when necessary
☐ Use as a course
☐ Use regularly
- daily,
- In the past month (30 days)
- In the past three months (90 days)
- In the past half a year (6 months)
- In the past year (12 months)
- Over a year

(Multiple answers possible in case of the use of several herbal medications)

12 a. Please mark the herbs that are an ingredient of teas, oils, tinctures, extracts, tablets, capsules or syrups. (Multiple answers possible)

☐ Aloe
☐ Hop
☐ St. John's wort
☐ Valerian
☐ Birch
☐ Plantain
☐ Ginkgo
☐ Cranberry
☐ Echinodermata
☐ Eleuthero
☐ Grapefruit
☐ Bird cherry
☐ Ginger
☐ Juniper
☐ Calendula
☐ Alder buckthorn
☐ Chamomile
☐ Garlic
☐ Liquorice
☐ Lime flowers
☐ Linseed
☐ Motherwort
☐ Evening primrose
☐ Urtica
☐ Oak
☐ Yarrow
☐ Peppermint
☐ Salvia
☐ Senna
☐ Onion
☐ Seabuckthorn
☐ Wormwood
☐ Hawthorn
☐ Meadowsweet
☐ Green tea
☐ Ginseng
☐ Coffee

13. Where do you get the information about the herbs and herbal medications?
(Multiple answers possible) Use the most appropriate answer Yes or No

Doctor	Pharmacist	Friends	Family	Advertisement	Personal choice	Other
Yes/No	Yes/No	Yes/No	Yes/No	Yes/No	Yes/No	Yes/No

14. Medical herbs and herbal medications are (please select most appropriate)

☐ Safe for use and harmless
☐ Can cause unwanted reaction, such as allergy
☐ Can influence the effect of other medication
☐ Safe use of medication is not important for me

15. When starting to use a new medical herb or herbal medication, do you check its side effects and allergic reactions?

Score	Very often	Often	Average	Rare	Very rare
	1	2	3	4	5

Thank you for taking your time to fill this questionnaire!
