# Supplementary material for: Safety Concerns Related to the Simultaneous Use of Prescription or Over-the-Counter Medications and Herbal Medicinal Products: Survey Results among Latvian Citizens
Source: Int J Environ Res Public Health. 2023 Aug 9;20(16):6551. doi: 10.3390/ijerph20166551 (PMC10454617; doi:10.3390/ijerph20166551)
Supplement: Supplementary file 1 [file ijerph-20-06551-s001.zip › S2. Ethics Commission.pdf]

RED CROSS MEDICAL
COLLEGE OF RĪGA
STRADIŅŠ UNIVERSITY

Approved by RSU RCMC director's
Order Nr. 1-8/92 of 14.10.2016.

Minutes Nr. 31

December 5, 2019

Meeting opened at 15:00
Meeting closed at 15:20

Agenda:
1. Review and evaluate RSU RCMC lecturer's Elita Ardava research documentation
2. Confirm the Ethics Commission decision

Meeting chaired by: Head of Ethics Commission Ilze Sauškina
Minutes taken by: Member of Ethics Commission Renāte Šukele
Participants: Deputy Head of Ethics Commission Ilze Celma

Documents submitted to the Commission:
1. Application
2. Questionnaire

Name of the research: The use of drugs, nutritional supplements and herbal medication

Research method: quantitative research

Research respondents: No less than 500 pharmacy clients of age 18 and above, who purchase drugs, nutritional supplements and herbal medication

Research base: Latvia pharmacies in different regions

Meeting participants' decision: After reviewing the submitted documents the commission decided that the research corresponds to the ethical requirements and it is allowed to carry out the research.

Chair of the meeting: Ilze Sauškina [signature]

Minute taker: Renāte Šukele [signature]
